# Supplementary material for: Designed active-site library reveals thousands of functional GFP variants
Source: Nat Commun. 2023 May 20;14:2890. doi: 10.1038/s41467-023-38099-z (PMC10199939; doi:10.1038/s41467-023-38099-z)
Supplement: Supplementary file 1 — Supplementary Information [file 41467_2023_38099_MOESM1_ESM.pdf]

Supplementary Information

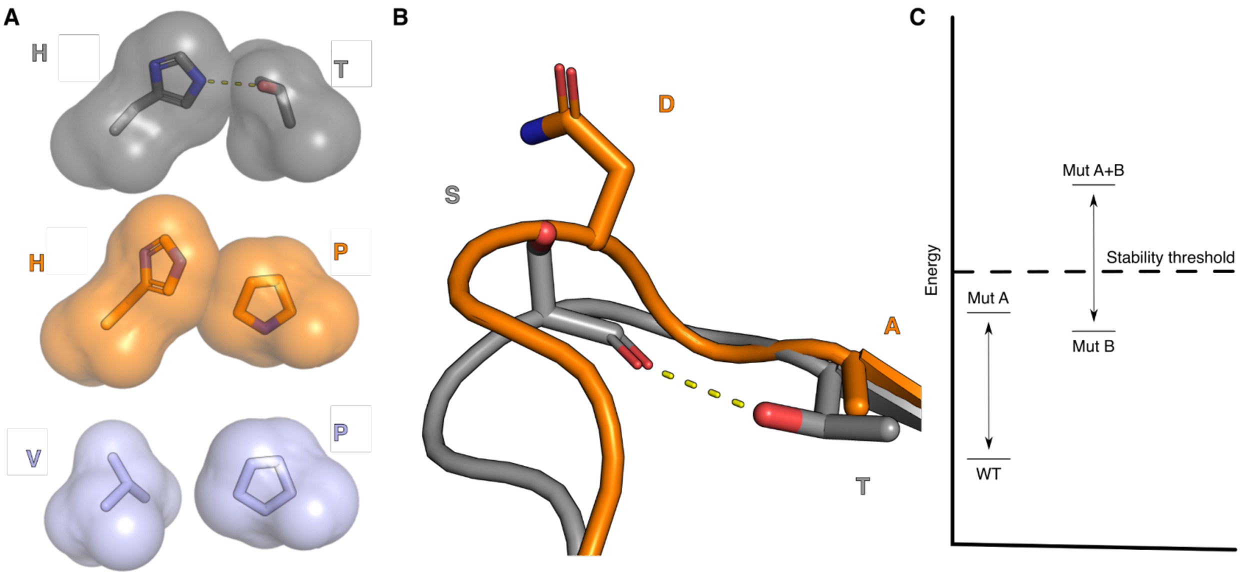

Supplementary Figure 1. Examples of direct and indirect epistasis. (**A**) In direct epistasis, interacting amino acids form favorable contacts (e.g., a hydrogen bond between the Thr and His residues). The double mutant Val/Pro pair is also favorable, but the point mutant Thr→Pro (middle) sterically overlaps with the His. (**B**) In indirect epistasis, a mutation (Thr→Ala) eliminates a hydrogen bond to the backbone (dashed line), leading to a conformational change across several non-interacting amino acids. This example is taken from a comparison of the structures of human and computationally designed variant acetylcholinesterase. Protein Data Bank entries 4EY4 and 5HQ3 are in gray and orange, respectively[60]. Showing positions 110 and 112. (**C**) Schematic explanation of stability-mediated interactions, the wild type (WT), mutant A, and mutant B are stable (below the stability threshold). The energy of the double mutant is a linear sum of the two energies of the two mutants, but the double mutant cannot be stably produced as its energy has crossed the threshold (marked by a dashed line)[40].

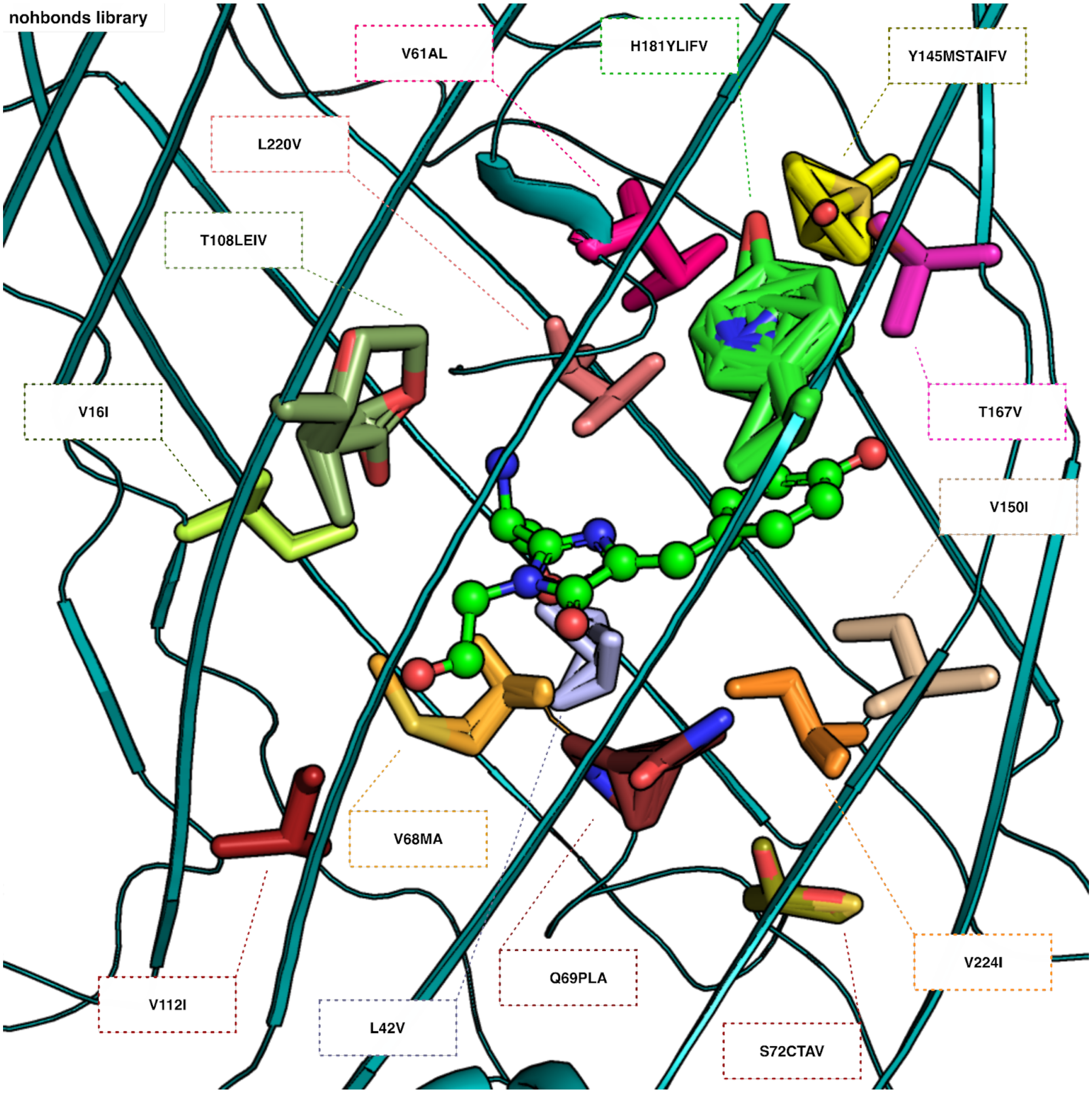

Supplementary Figure 2. Structural overview of the nohbonds library. All mutations are overlayed in stick representation, colored by position. The total library size is 11,059,200 designs.

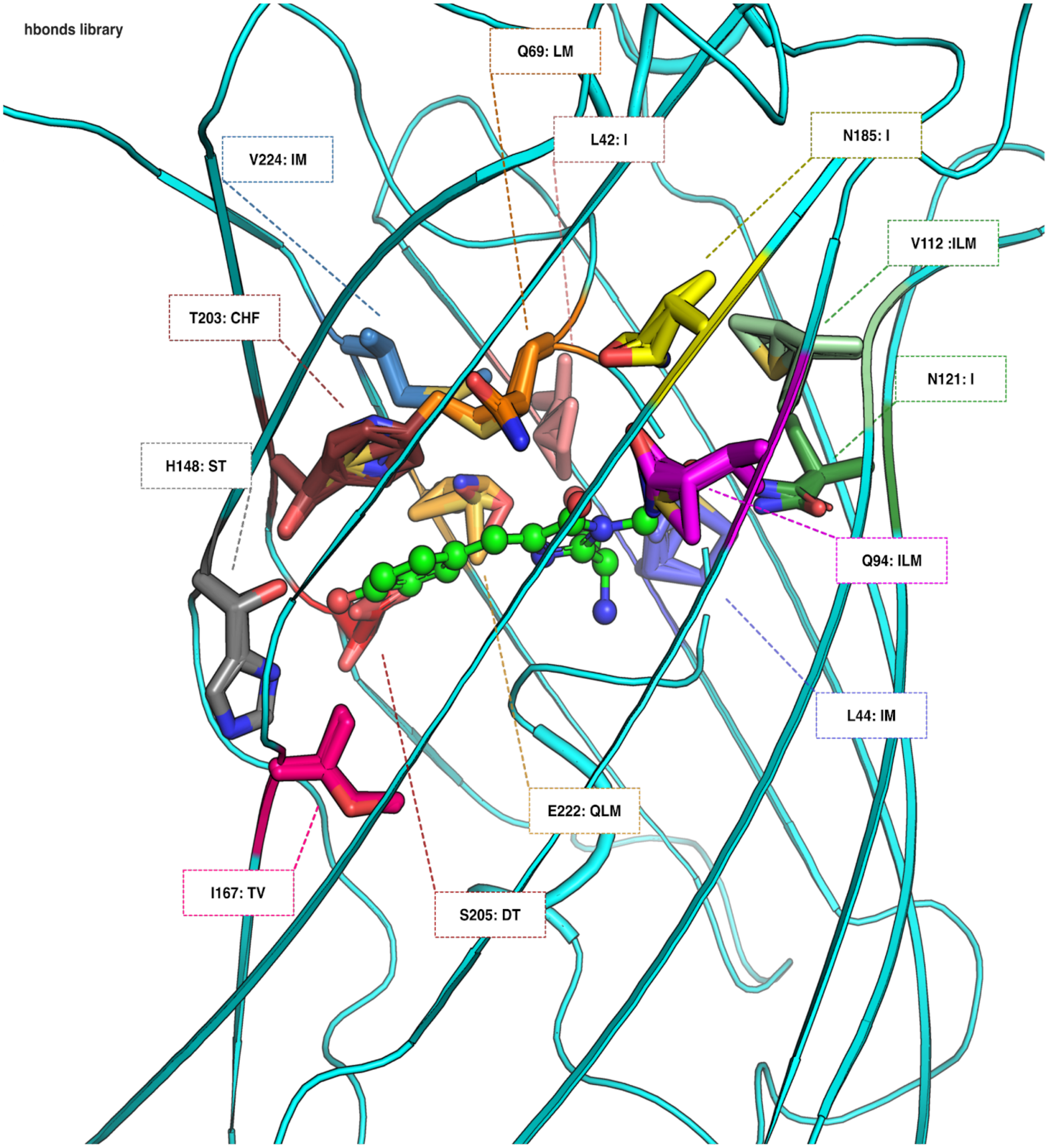

Supplementary Figure 3. Structural overview of the hbonds library. All mutations are overlayed in stick representation, colored by position. The total library size is 933,120 designs.

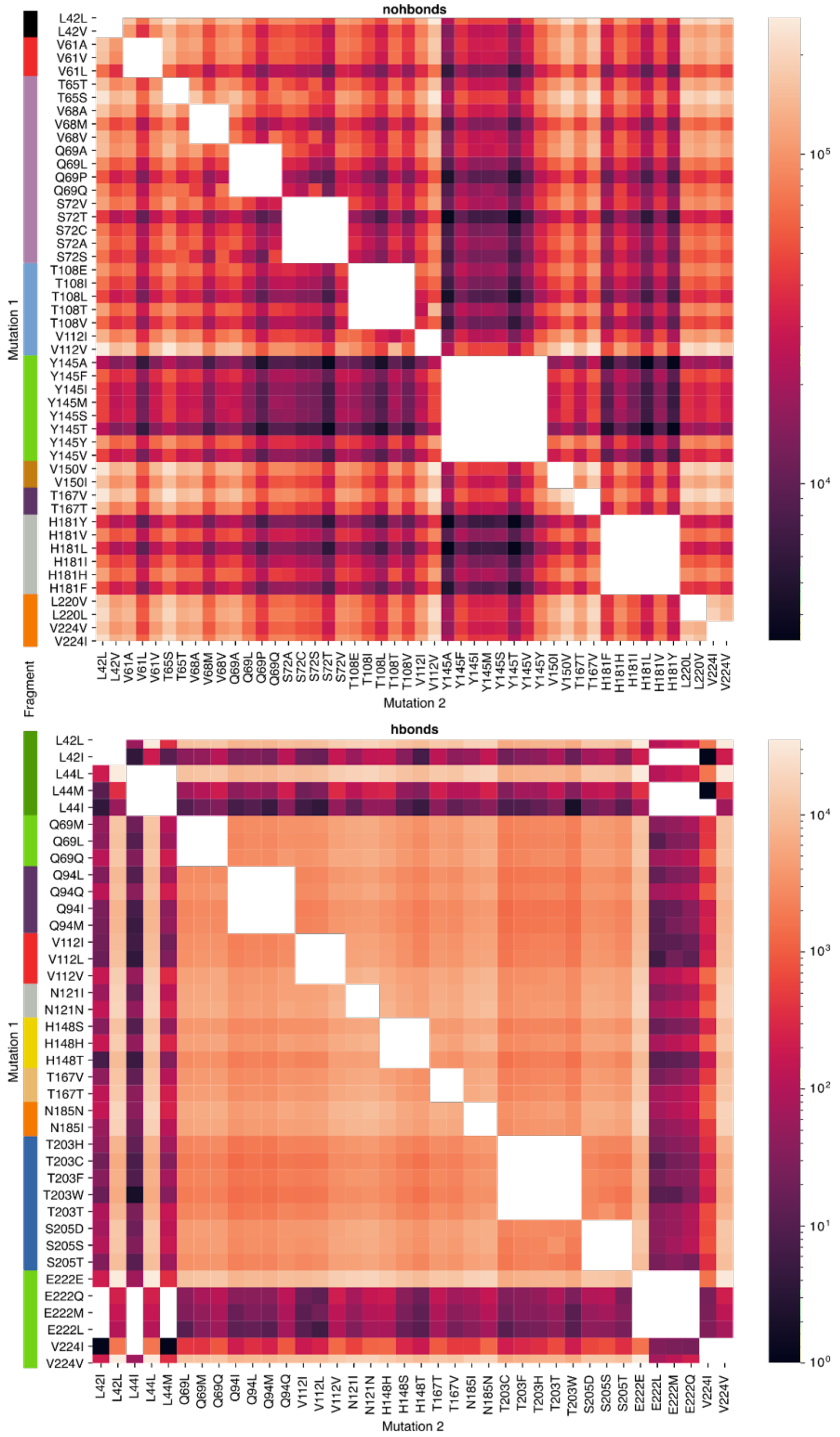

Supplementary figure 4. Golden Gate assembly validation. Both libraries were cloned using Golden-Gate assembly. The number of occurrences of each pair of mutations is shown as a

heat map. Sequence positions that were on the same oligonucleotide are marked with a continuous colored bar on the left. There is no obvious linkage between any pair of mutations, which means that mutations are uniformly represented. Additionally, all single and double mutations were present in the nohbonds library. The hbonds library suffered from small diversity at the edges (first and last oligonucleotides), and thus not all pairs of mutations are represented. Mutations in the same position are masked in white as a single sequence cannot have two mutations at the same position. Source data are provided as a Source Data file.

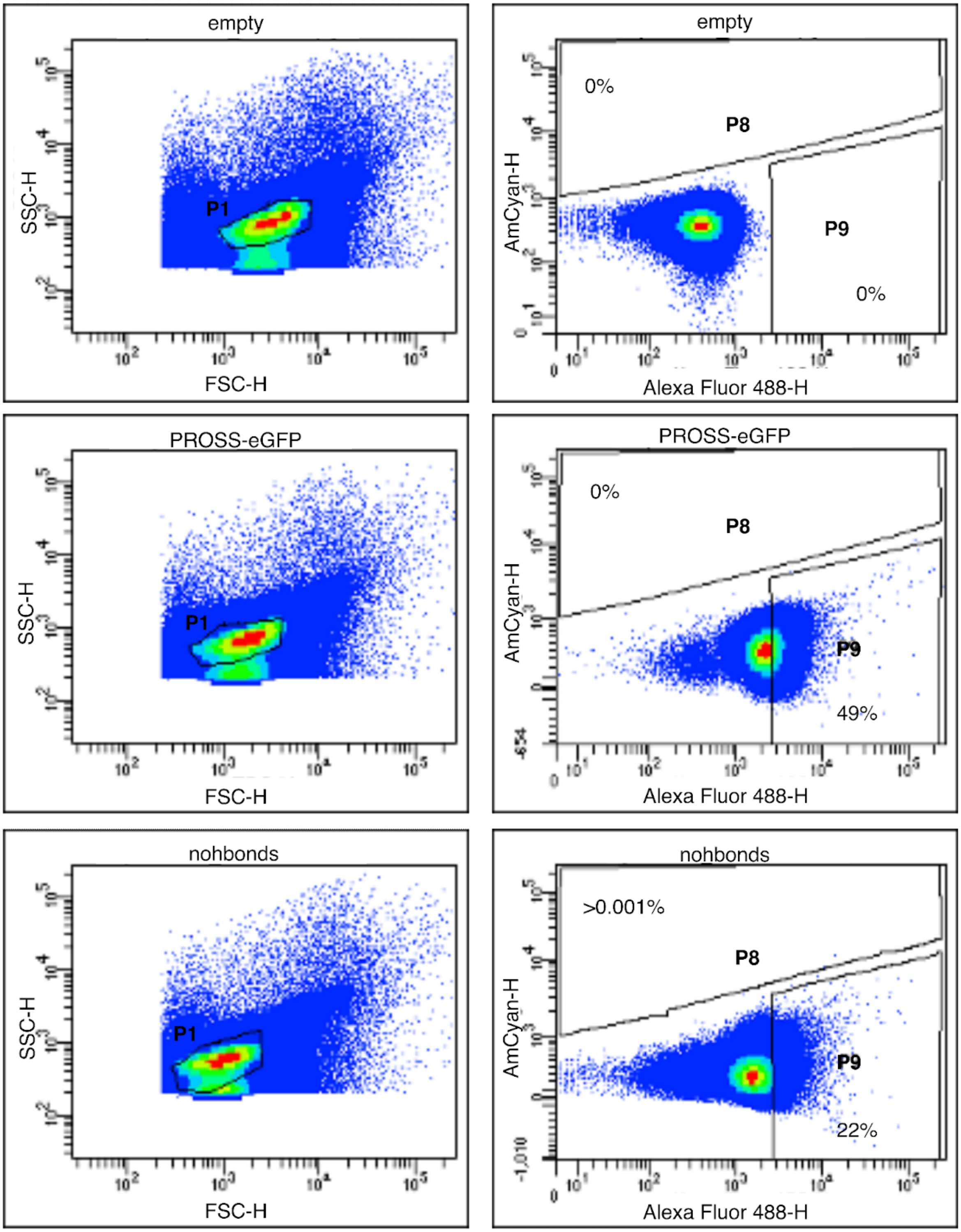

Supplementary Figure 5. Library sorting gates. The hbonds and nohbonds libraries were sorted for excitation at both 405 and 488, with emission at 530 and 525, respectively. Alexa Fluor 488 measures excitation and emission at 488 and 530 nm, respectively. AmCyan

measures excitation and emission at 405 and 525 nm, respectively. Each panel shows only 10,000 events.

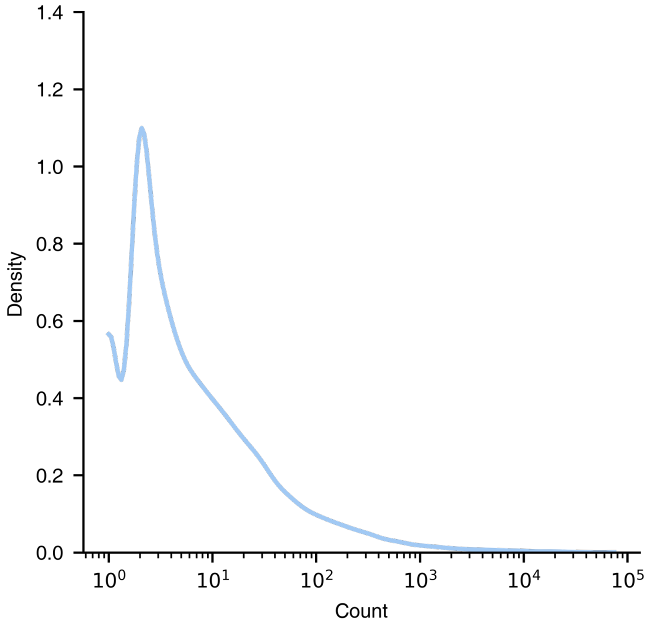

Supplementary Figure 6. Deep-sequencing counts across the sorted samples. Number of times each unique sequence in all sorted samples was found in the deep-sequencing data. Source data are provided as a Source Data file.

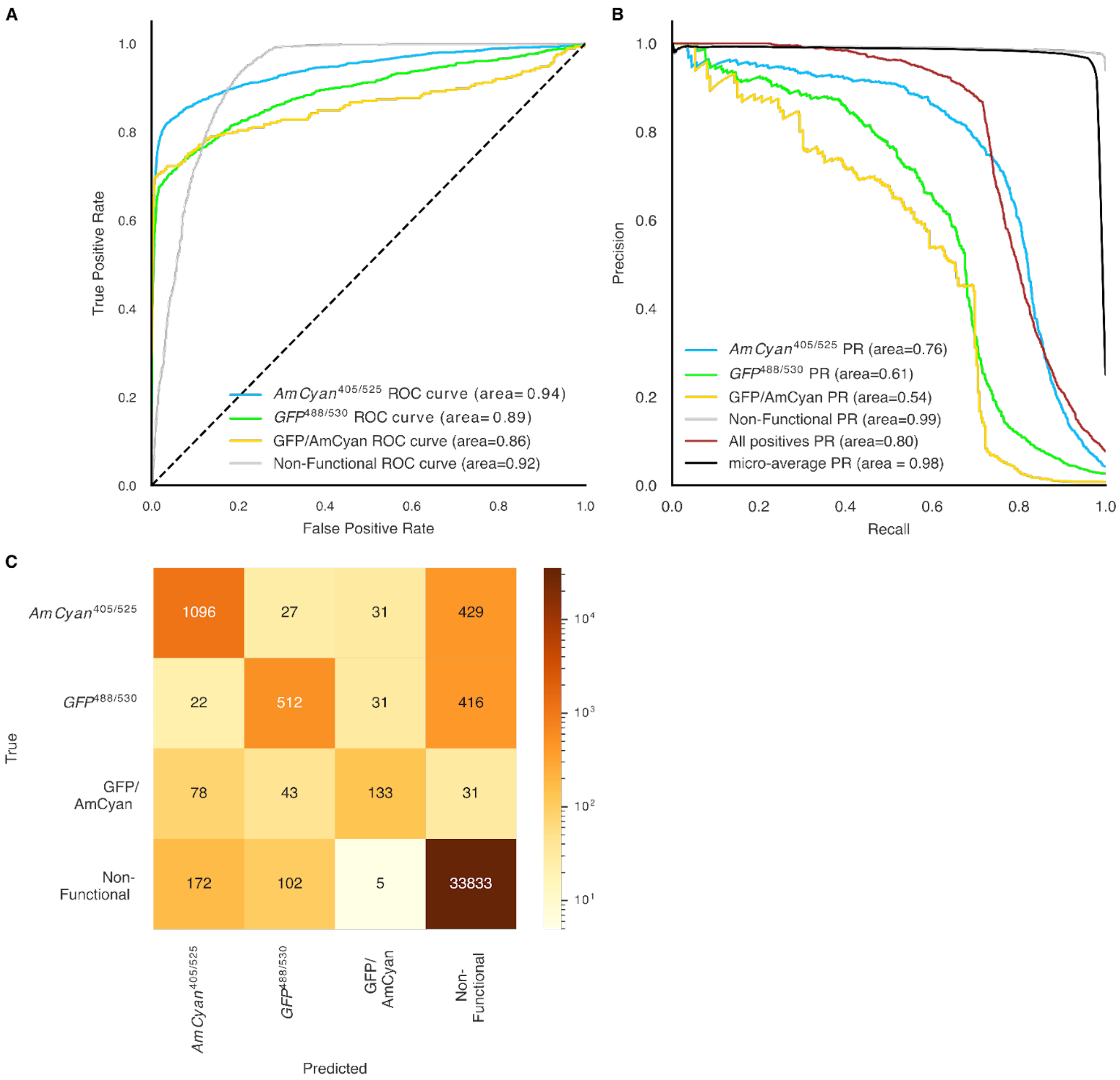

Supplementary Figure 7. Random forest prediction analysis. (A) Receiver Operating Characteristic (ROC) analysis of classification accuracy to all four classes. (B) Precision-Recall analysis for all four classes. “All positives” refers to only the functional classes, and “micro-average” refers to a sliding window that measures the average precision across all classes. The area under the curve (AUC) and average precision (AP) are reported for the ROC and precision-recall analysis, respectively. (C) A confusion matrix of prediction results. All analyses were conducted on an independent test set. The random forest is fairly accurate in determining whether a given sequence is functional, and is somewhat less accurate in assigning a specific functional classification (GFP, AmCyan, or GFP/AmCyan). Source data are provided as a Source Data file.

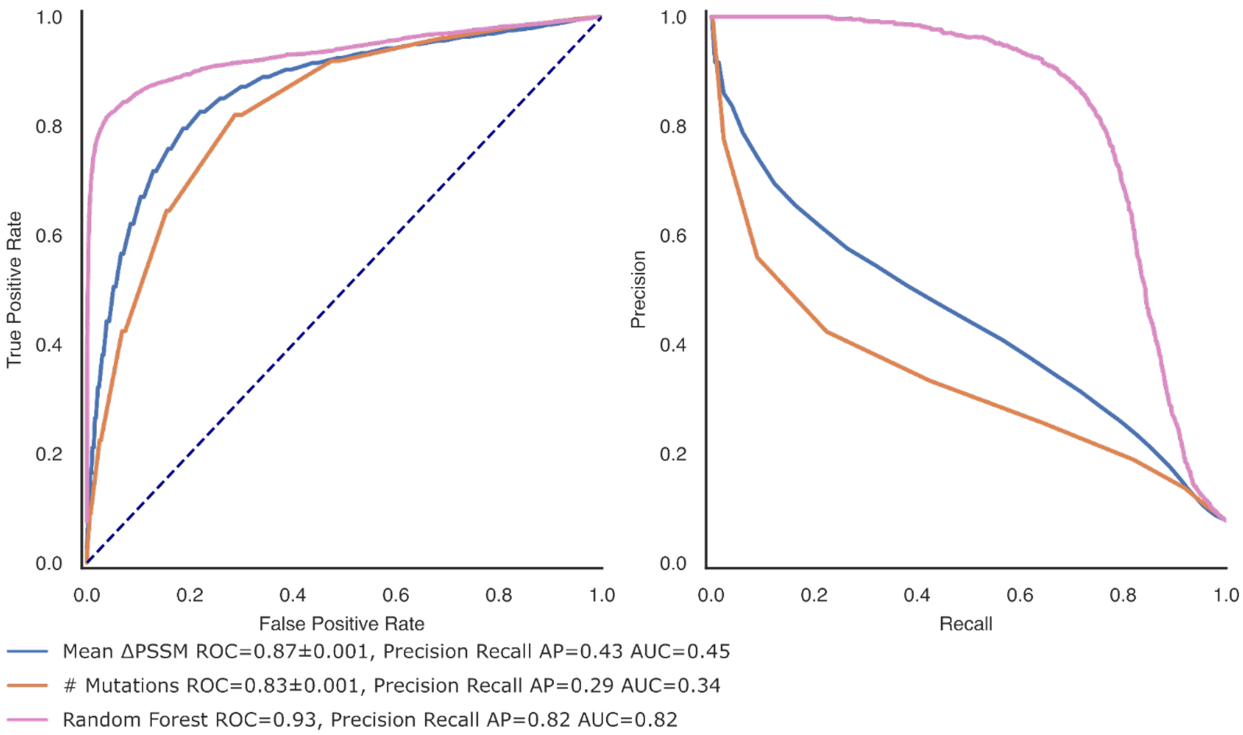

Supplementary Figure 8. Mean ΔPSSM and number of mutations predict design functionality.

Prediction accuracy analysis for mean ΔPSSM and number of mutations, receiver operator curve (ROC, left), and Precision-Recall curve (right). The area under the curve (AUC) and average precision (AP) are reported for the ROC and precision-recall analysis, respectively. Source data are provided as a Source Data file.

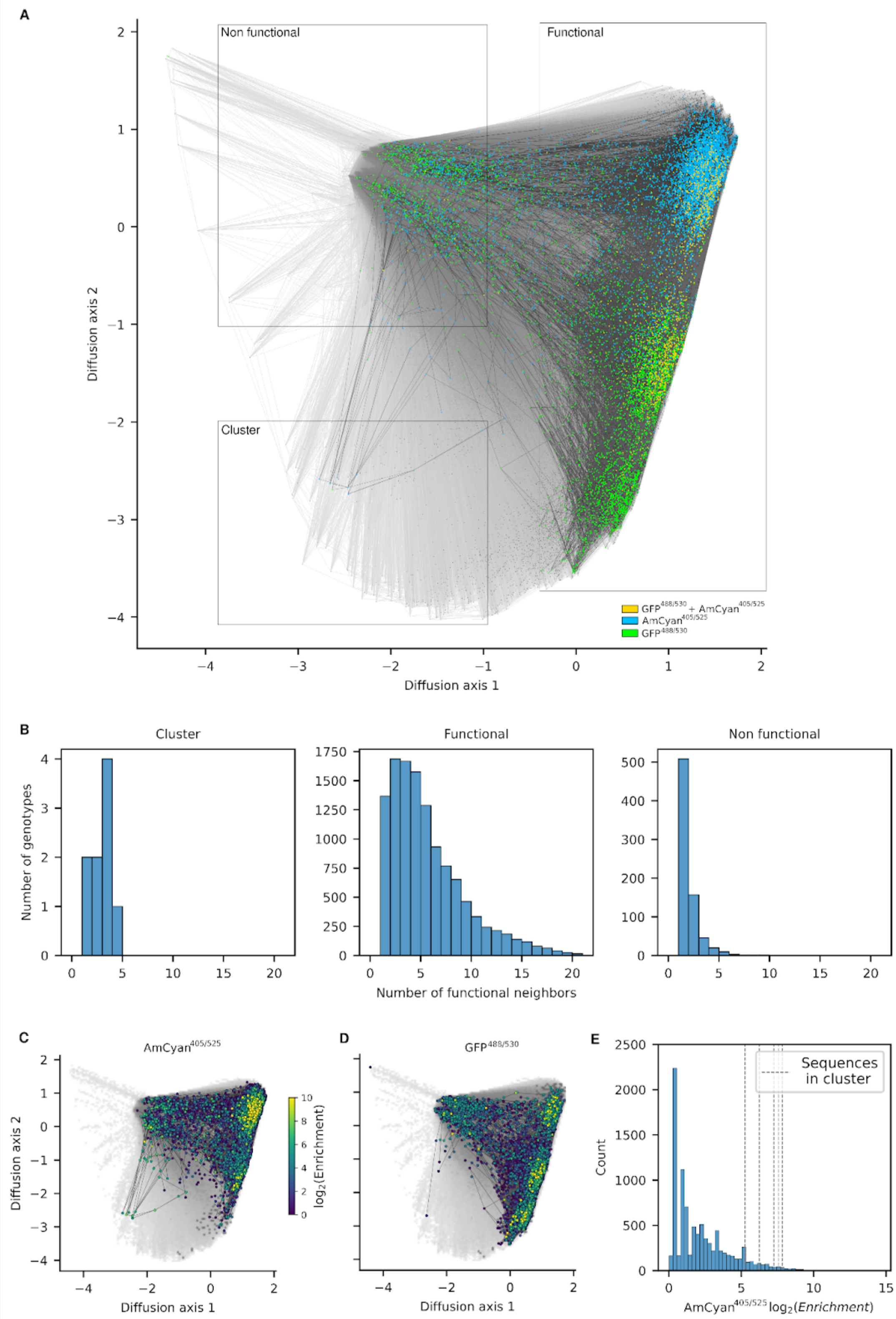

Supplementary Figure 9. Fitness landscape visualizations showing experimentally enriched sequences. (A) Low dimensional visualization of the sequence-function relationship

predicted by the random forest model. Sequences are highlighted in different colors according to whether they are detected to be enriched in the $GFP^{488/530}$ (green), $AmCyan^{405/525}$ (blue) or both channels (orange) in the high-throughput data. Dark lines join experimentally enriched genotypes that are separated by single amino acid substitutions. **(B)** Degree distributions for genotypes located at different regions of the visualization as observed directly in the experimental data: in the minor cluster, the main set of functional sequences, and the set of genotypes that the RF predicted to be non-functional. Non-functional genotypes tend to be more poorly connected in the graph of experimentally determined sequences than those in the main set of functional sequences (two-sided Mann-Whitney U test, statistic=7576740.5, p-value=$4.46 \cdot 10^{-273}$), further suggesting that, on average, they are false positives correctly smoothed by the RF. The small cluster of functional sequences predicted by the RF shows a higher connectivity than the set of non-functional sequences (two-sided Mann-Whitney U test, statistic=5106.0, p-value=0.0008), providing an additional line of evidence for their functionality. **(C, D)** Low dimensional visualization of the sequence-function relationship predicted by the random forest model. Overlaid sequences represent the sequences that were enriched in the experimental data. The color scale from purple to yellow represents the $\log_2$(Enrichment) in the sorted vs unsorted fractions for $AmCyan^{405/525}$ (C) and $GFP^{488/530}$ (D). Sequences with higher enrichment values are represented on top and the color scale was truncated at $\log_2$(Enrichment)=10. **(E)** Histogram of the $\log_2$(Enrichment) in the sorted vs unsorted fractions for $AmCyan^{405/525}$. Vertical lines show the values for sequences in the Cluster highlighted in (A). Source data are provided as a Source Data file.

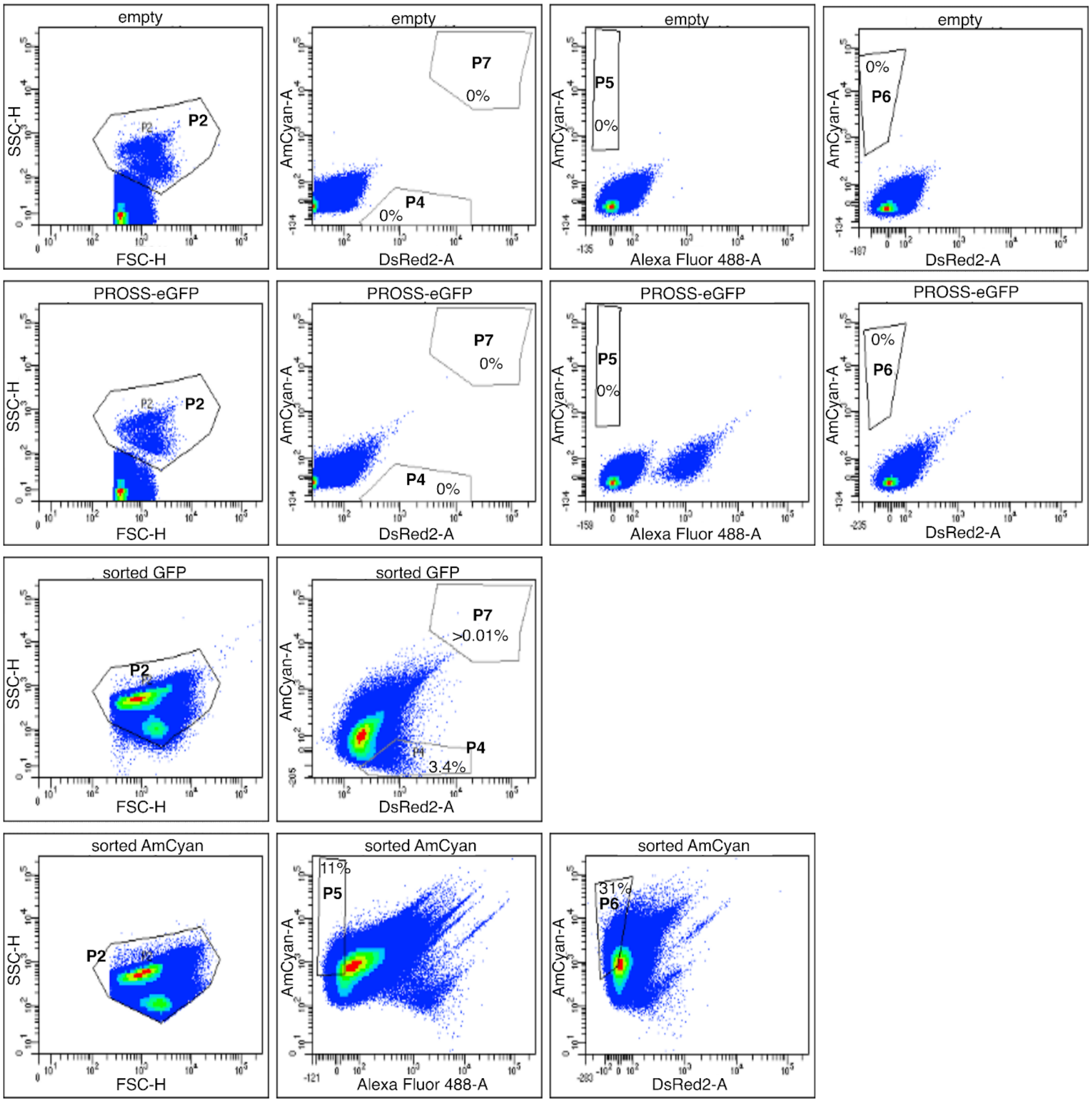

Supplementary Figure 10. Screen for designs with shifted fluorescence spectra. We sorted the GFP and AmCyan pre-sorted libraries for designs that exhibit spectral shifts compared to PROSS-eGFP and eGFP. From top to bottom: empty vector as a negative control, PROSS-eGFP as a positive control, the library sorted for GFP fluorescence and the library sorted for AmCyan fluorescence. Alexa Fluor 488 measures excitation and emission at 488 and 530 nm, respectively. AmCyan measures excitation and emission at 405 and 525 nm, respectively. DsRed2 measures excitation and emission at 561 and 582 nm, respectively.

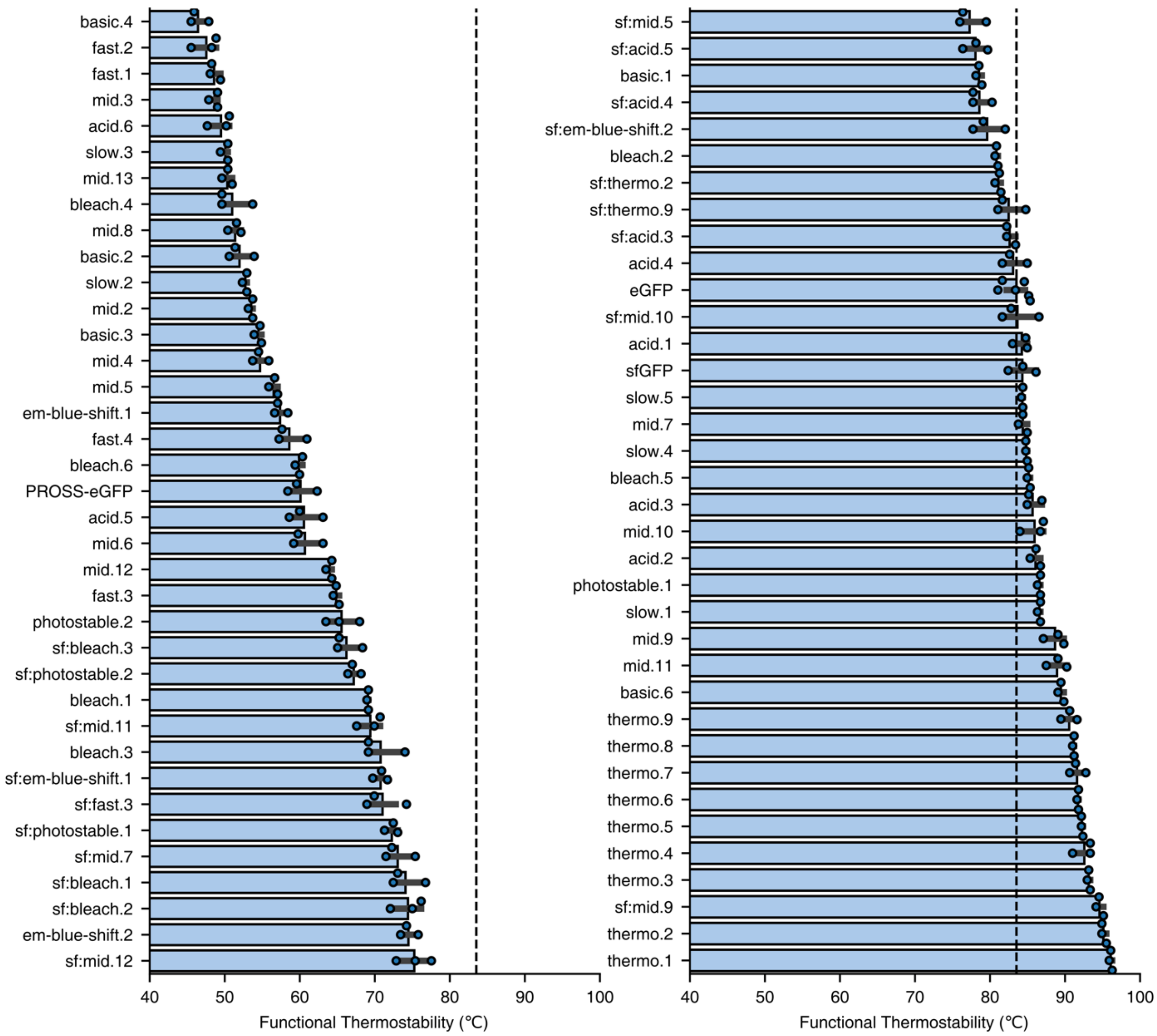

Supplementary Figure 11. Functional thermostability of selected designs. Functional thermostability is the temperature at which fluorescence is at 50% of the max. The dashed line marks the thermostability of eGFP. Data are presented as mean ± standard deviation. *n*=3 biologically independent samples were tested. Source data are provided as a Source Data file.

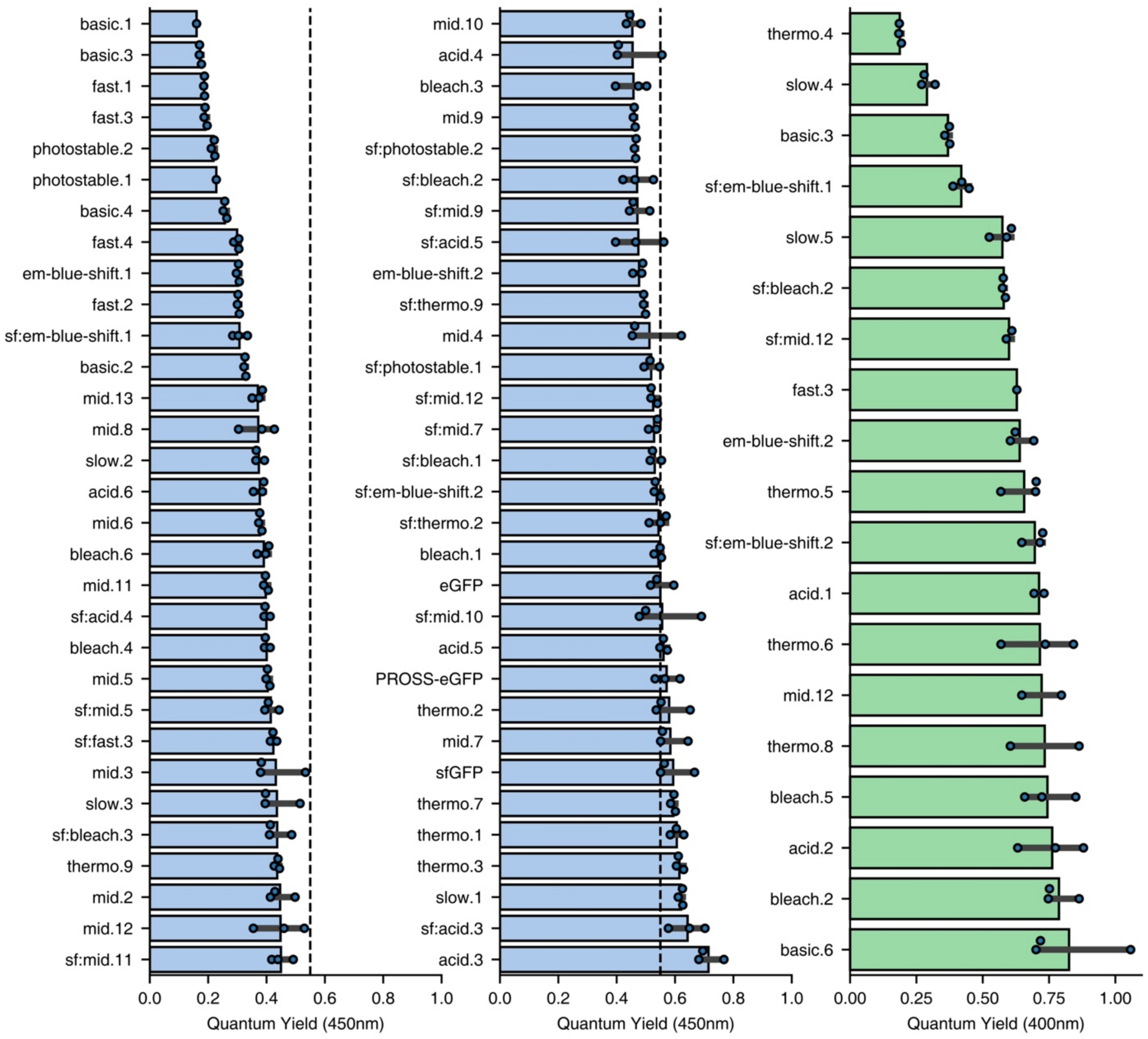

Supplementary Figure 12. Quantum yield measurement for all designs with excitation at either 400 or 450 nm. The dashed line marks the quantum yield of eGFP (which is not excited at 400nm). Data are presented as mean ±standard deviation. *n*=3 biologically independent samples were tested. Source data are provided as a Source Data file.

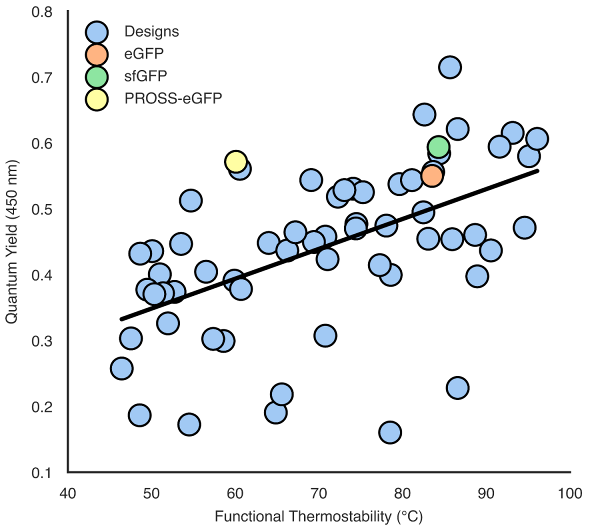

Supplementary Figure 13. Functional thermostability and quantum yield are correlated. Two-sided Pearson's r=0.53 (p-value=8.37x10^{-6}) between functional thermostability and quantum yield at 450nm excitation. Source data are provided as a Source Data file.

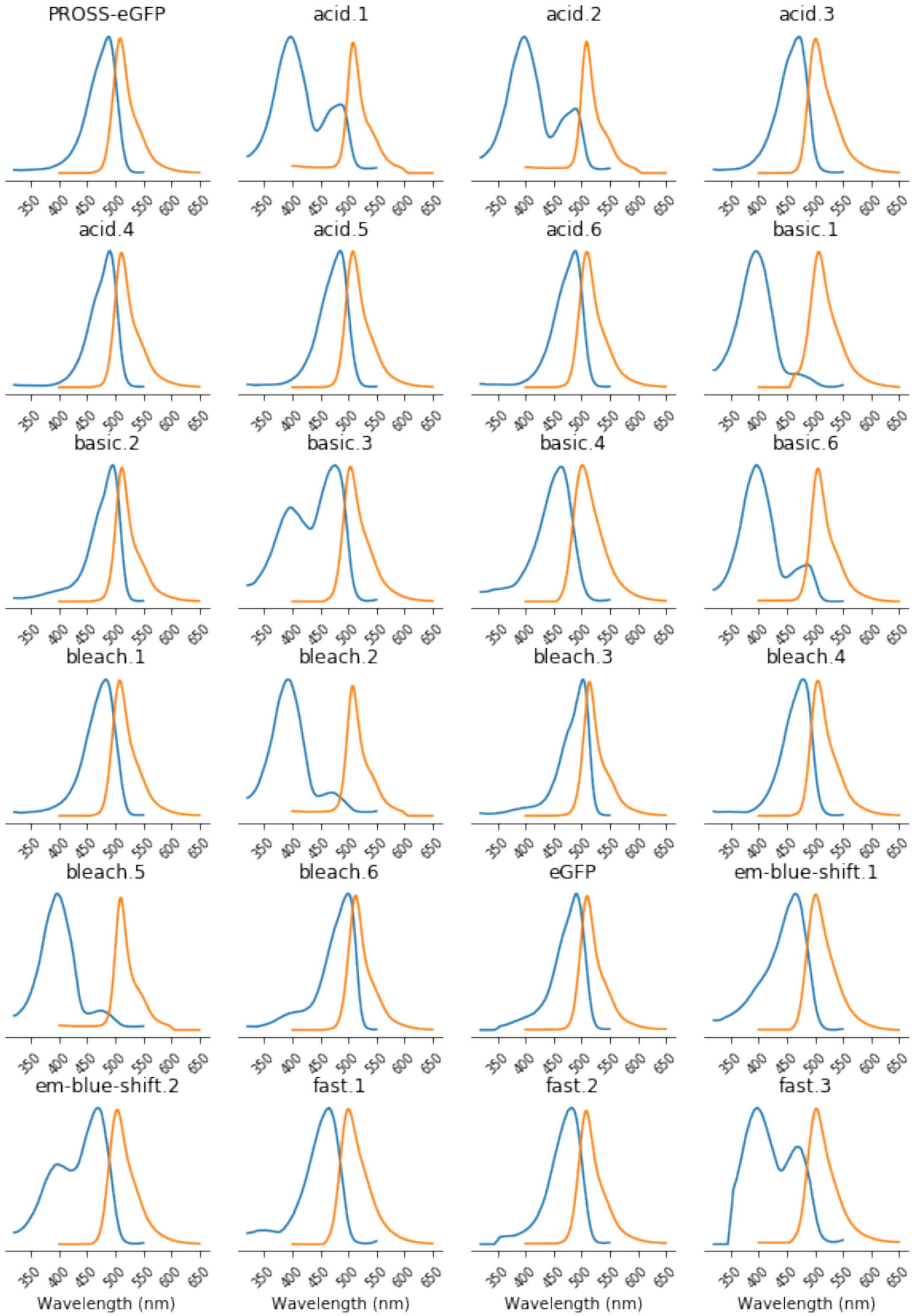
PROSS-eGFP
acid.1
acid.2
acid.3
acid.4
acid.5
acid.6
basic.1
basic.2
basic.3
basic.4
basic.6
bleach.1
bleach.2
bleach.3
bleach.4
bleach.5
bleach.6
eGFP
em-blue-shift.1
em-blue-shift.2
fast.1
fast.2
fast.3
350 400 450 500 550 600 650
Wavelength (nm)

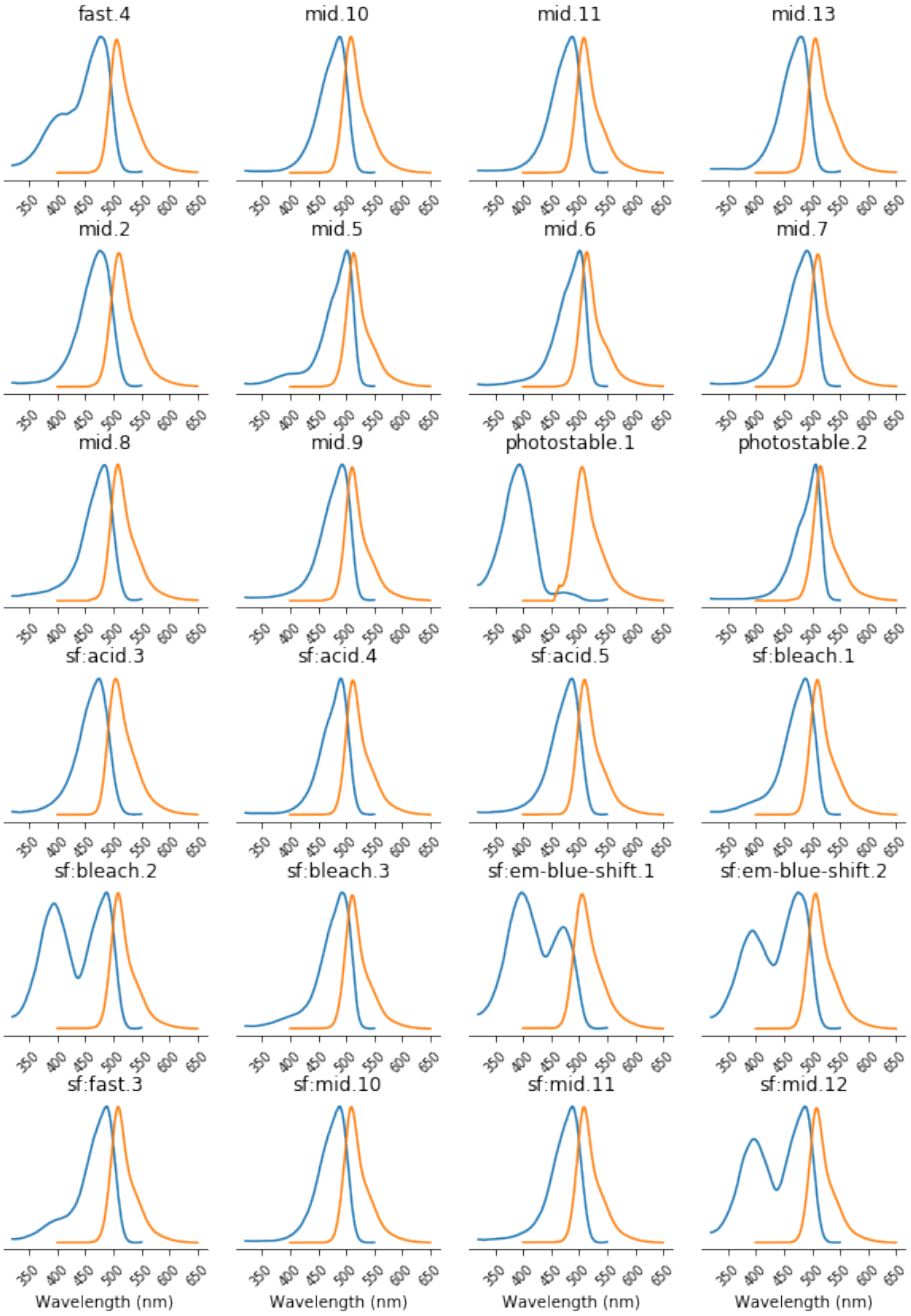
fast.4
mid.10
mid.11
mid.13
mid.2
mid.5
mid.6
mid.7
mid.8
mid.9
photostable.1
photostable.2
sf:acid.3
sf:acid.4
sf:acid.5
sf:bleach.1
sf:bleach.2
sf:bleach.3
sf:em-blue-shift.1
sf:em-blue-shift.2
sf:fast.3
sf:mid.10
sf:mid.11
sf:mid.12
350 400 450 500 550 600 650
Wavelength (nm)

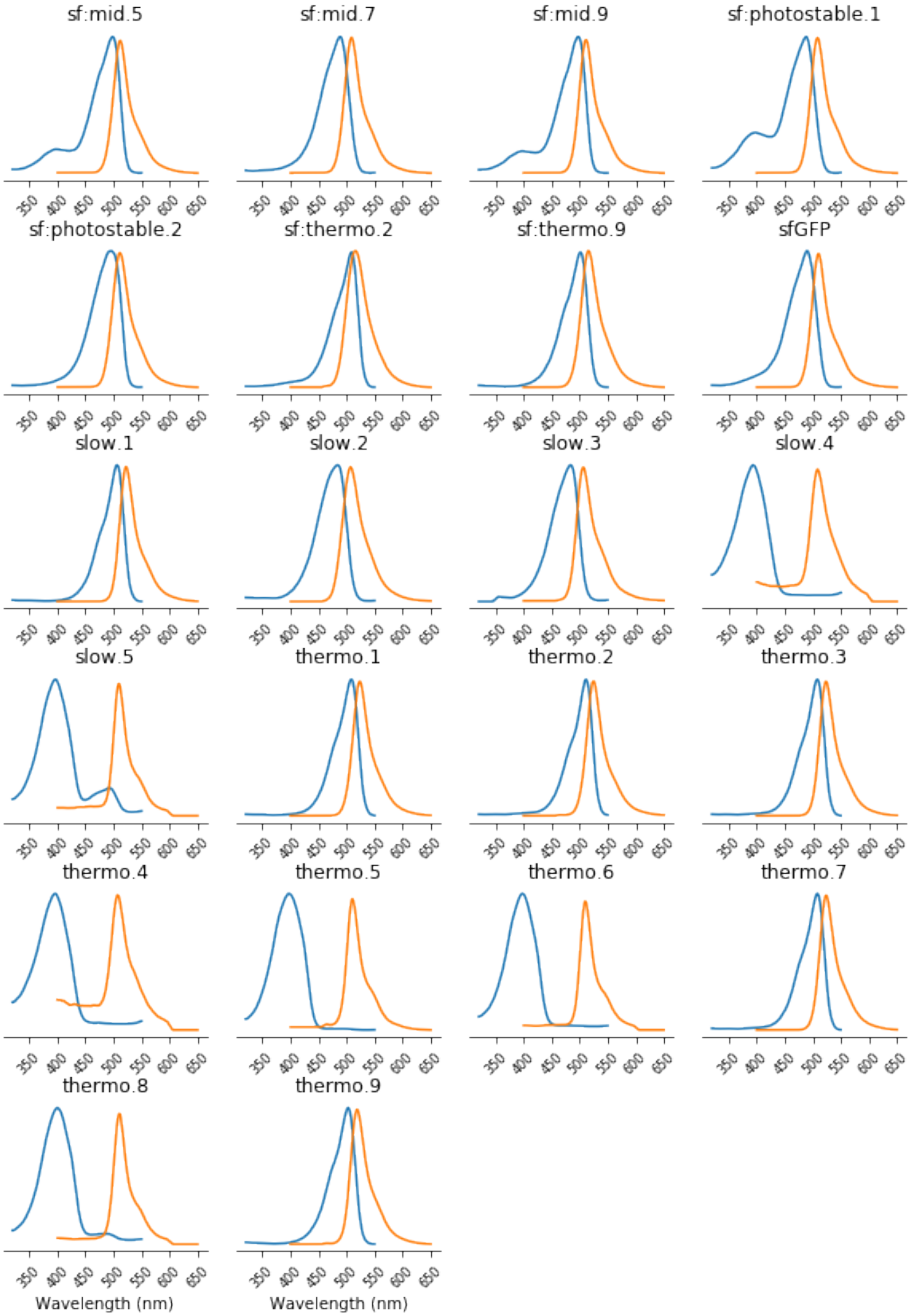
sf:mid.5
sf:mid.7
sf:mid.9
sf:photostable.1
sf:photostable.2
sf:thermo.2
sf:thermo.9
sfGFP
slow.1
slow.2
slow.3
slow.4
slow.5
thermo.1
thermo.2
thermo.3
thermo.4
thermo.5
thermo.6
thermo.7
thermo.8
thermo.9
350 400 450 500 550 600 650
Wavelength (nm)
Wavelength (nm)

Supplementary Figure 14. Spectral properties of all tested designs. Excitation and emission spectra are shown in blue and orange colors, respectively. Source data are provided as a Source Data file.

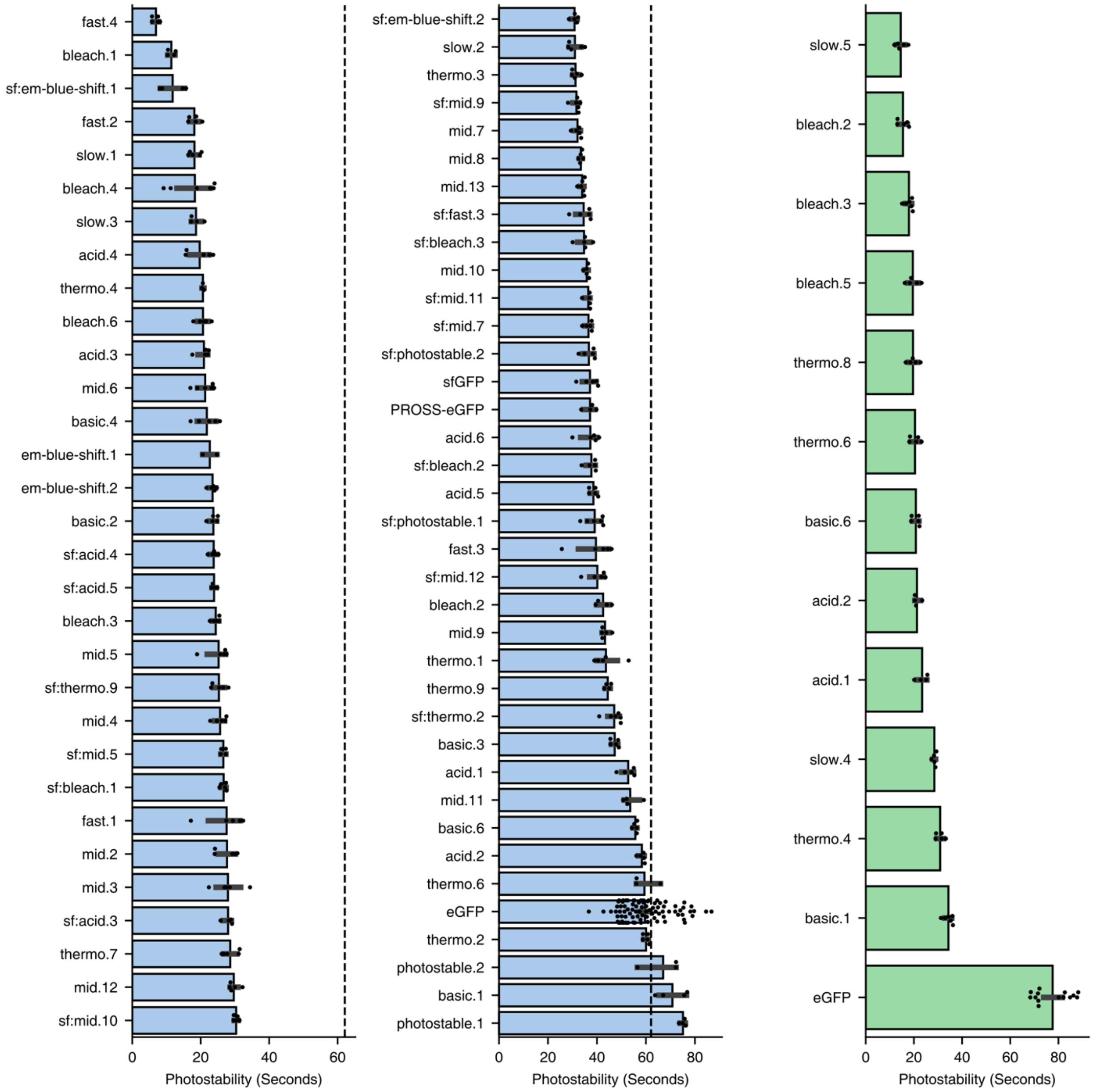

Supplementary Figure 15. Photobleaching measurement of all selected designs. Shows average and standard deviation of at least three independent measurements of photobleaching (Methods). The dashed line marks the eGFP. Data are presented as mean ± standard deviation. *n*=3 biologically independent samples were tested. Source data are provided as a Source Data file.

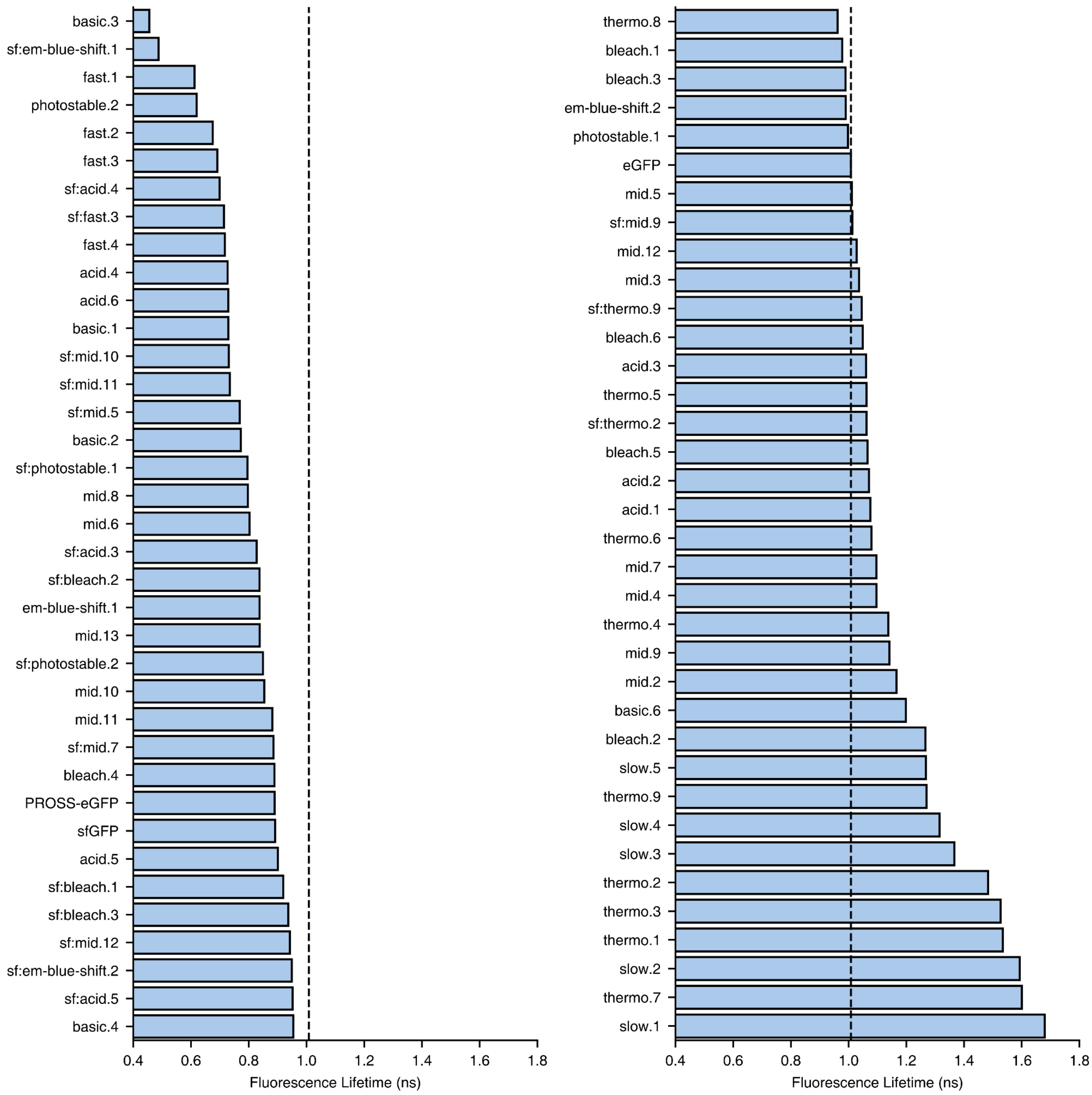

Supplementary Figure 16. Fluorescence lifetime measurements of all tested designs. Bars depict the weighted average of bi-exponential fit to lifetime measurements. The dashed line marks the lifetime of eGFP. Data are presented as mean ±standard deviation. One biological sample was used for fluorescence lifetime measurements due to numerous technical repeats and high costs. Source data are provided as a Source Data file.

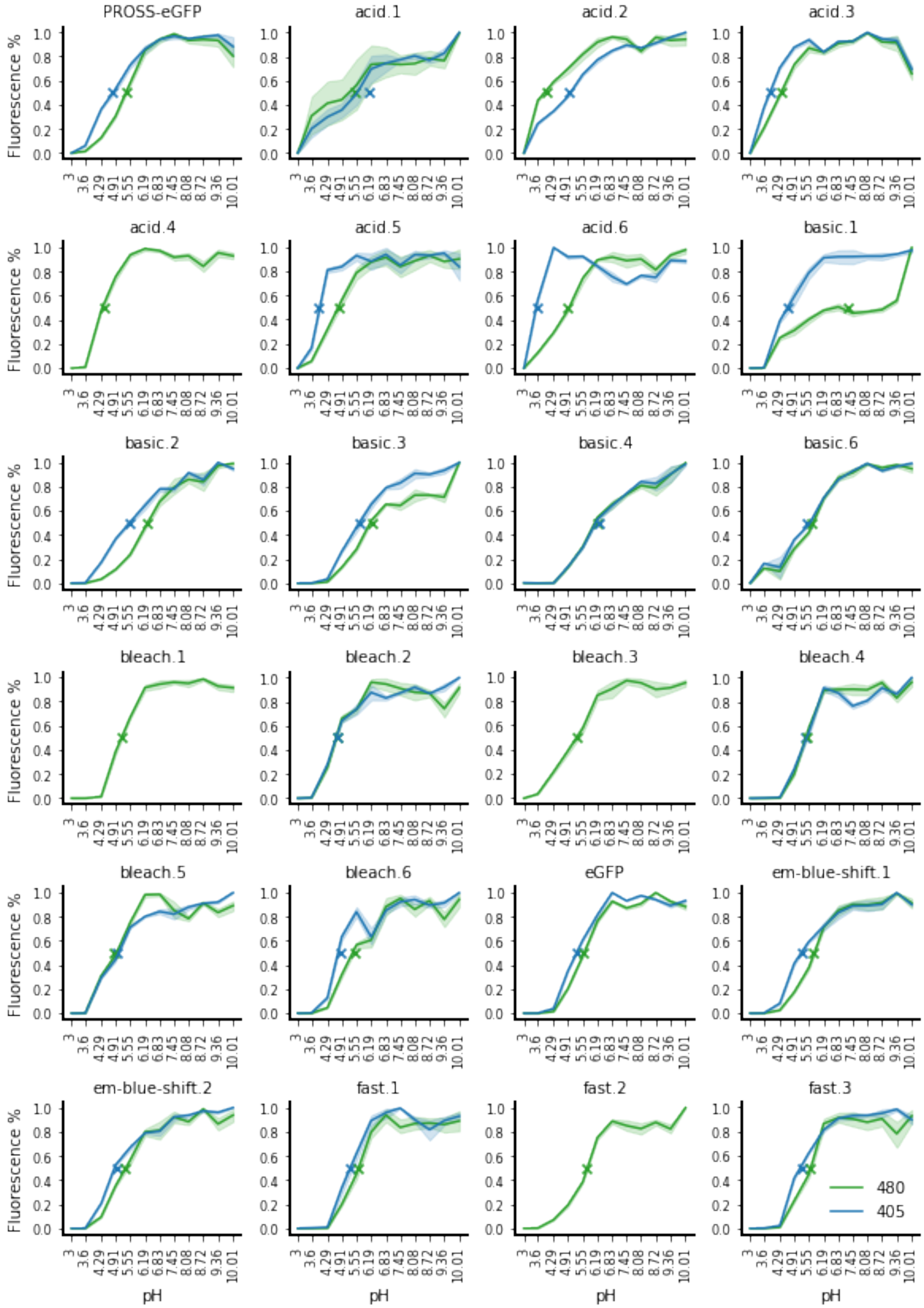
PROSS-eGFP
acid.1
acid.2
acid.3
acid.4
acid.5
acid.6
basic.1
basic.2
basic.3
basic.4
basic.6
bleach.1
bleach.2
bleach.3
bleach.4
bleach.5
bleach.6
eGFP
em-blue-shift.1
em-blue-shift.2
fast.1
fast.2
fast.3
Fluorescence %
0.0
0.2
0.4
0.6
0.8
1.0
3
3.6
4.29
4.91
5.55
6.19
6.83
7.45
8.08
8.72
9.36
10.01
pH
480
405

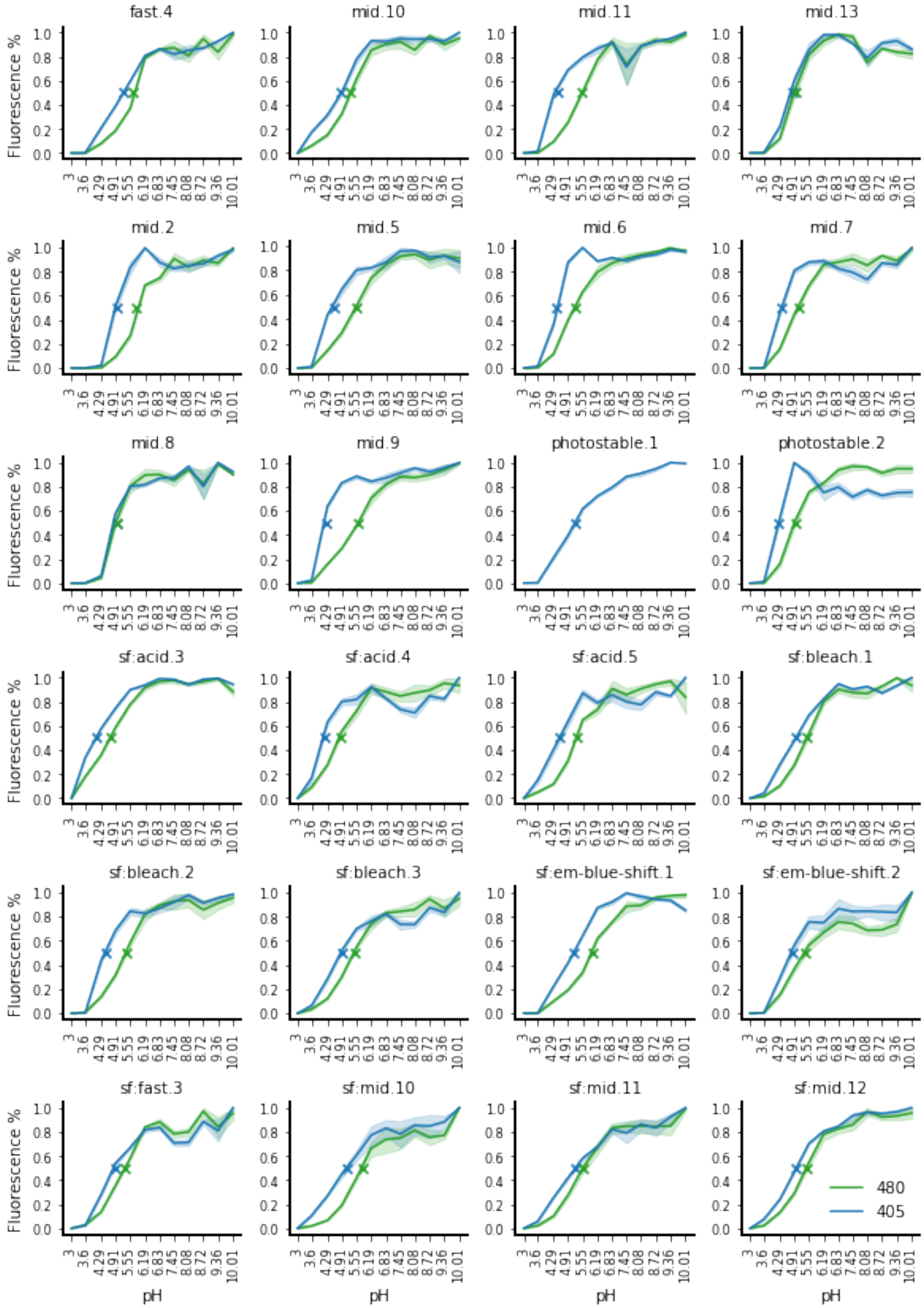

fast.4
mid.10
mid.11
mid.13
mid.2
mid.5
mid.6
mid.7
mid.8
mid.9
photostable.1
photostable.2
sf:acid.3
sf:acid.4
sf:acid.5
sf:bleach.1
sf:bleach.2
sf:bleach.3
sf:em-blue-shift.1
sf:em-blue-shift.2
sf:fast.3
sf:mid.10
sf:mid.11
sf:mid.12
Fluorescence %
0.0 0.2 0.4 0.6 0.8 1.0
3 3.6 4.29 4.91 5.55 6.19 6.83 7.45 8.08 8.72 9.36 10.01
pH
480
405

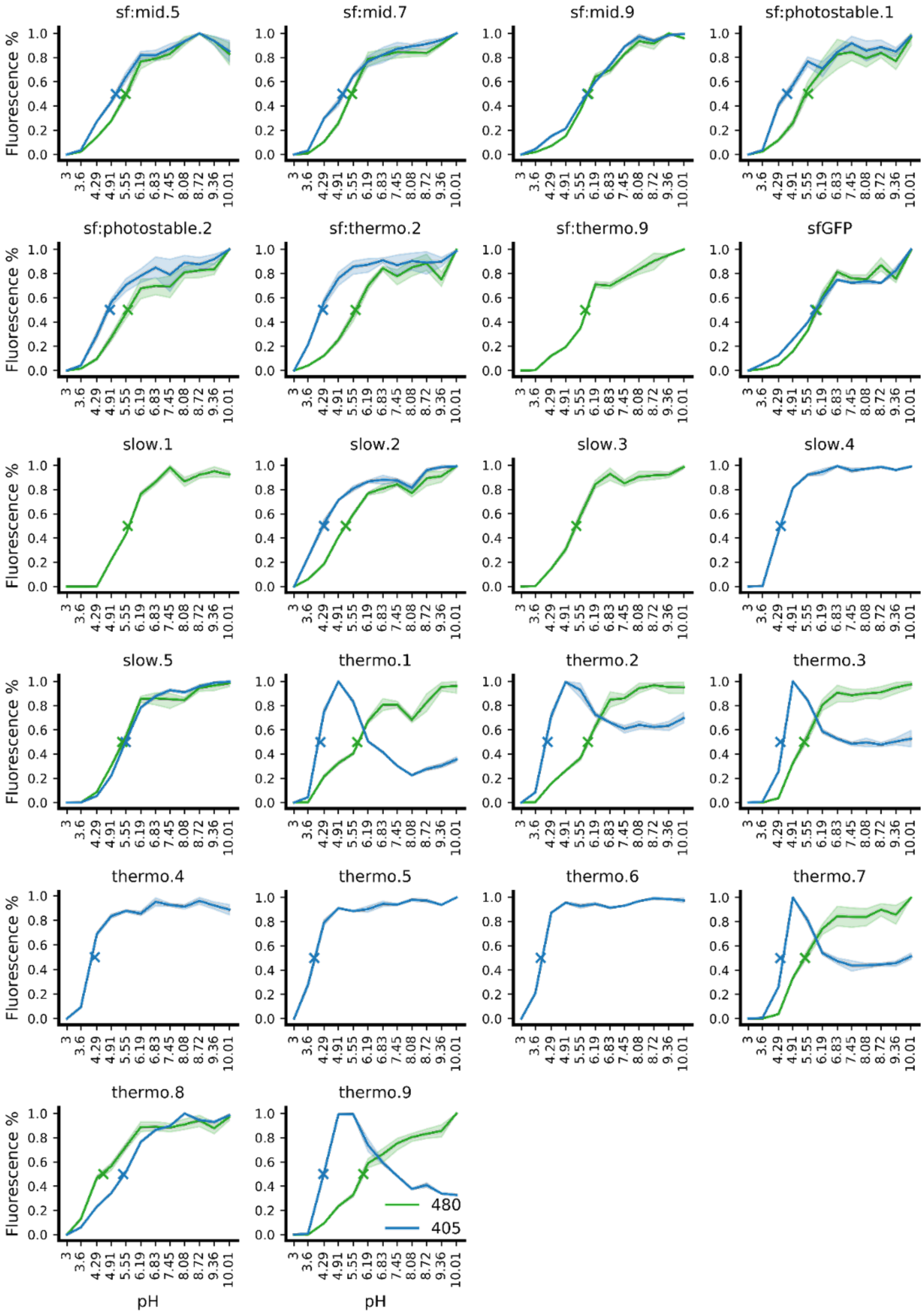

Supplementary Figure 17. pH sensitivity profiles. The normalized fluorescence of each design is shown as a function of buffer pH. Green and blue lines refer to excitation at 480 and 405 nm,

respectively. The pKa is the pH at which fluorescence is at 50% of the maximum, annotated by an “X”. A confidence interval of 95% is shown. Source data are provided as a Source Data file.

		Experimental	
		Functional	Non-functional
Deep-sequencing analysis	Functional	45	1
	Non-functional	5	11

Supplementary Table 1. Predictive values for the deep-sequencing data analysis. 62 designs were individually selected directly from FACS sorts and tested for fluorescence. These were used to calibrate the thresholds for the deep-sequencing analysis.

Dataset	A	B	RMSE
RF	0.0253	0.269	0.0134
NGS	0.223	0.134	0.0203
avGFP	0.184	0.191	0.0428
amacGFP	0.766	0.0427	0.0243
cgreGFP	0.542	0.518	0.0233
ppluGFP	0.334	0.248	0.0199

Supplementary Table 2. Overall epistasis and deleteriousness. Fitted parameters for all six datasets as shown in Figure 2C and D.

	Precision	Recall	f1-score	support
GFP	0.8	0.69	0.74	1583
AmCyan	0.75	0.52	0.62	981
GFP/AmCyan	0.67	0.47	0.55	285
Non-Functional	0.97	0.99	0.98	34112
accuracy			0.96	36961
macro avg	0.8	0.67	0.72	36961
weighted avg	0.96	0.96	0.96	36961

Supplementary Table 3. Classification accuracy metrics for the random forest. Conducted using an independent test set.

L42	V68	Q69	S72	T108	V112	Y145	T167	H181	L220	V224	Functional class	Enrichment (log2)
V	A	A	T	E	V	Y	T	H	V	I	AmCyan	7.2
V	A	A	T	E	V	M	T	H	V	I	AmCyan	7.6
V	A	A	T	E	V	F	T	H	V	I	AmCyan	5.2
V	A	A	T	E	V	Y	V	H	V	I	AmCyan	7.8
V	A	A	T	E	V	Y	T	H	L	I	AmCyan	6.2
V	A	A	T	E	V	I	T	H	V	I	AmCyan	5.2
V	A	A	T	E	V	Y	T	L	V	I	AmCyan	5.2
V	A	A	T	E	V	M	V	L	V	I	AmCyan	5.2
V	A	A	T	E	I	Y	T	H	V	I	GFP	1.3

Supplementary Table 4. Enrichment values for the highly connected cluster.

Source	Functional class	# designs tested	# functional designs
Deep-sequencing data	GFP (488/530nm)	10	6 (60%)
	AmCyan (405/525nm)	10	8 (80%)
	GFP & AmCyan	4	3 (75%)
	Total	24	17 (71%)
	Max number of mutations	6	0
	Designs of special interest	3	1 (33%)
Random forest predictions	GFP (488/530nm)	5	4 (80%)
	AmCyan (405/525nm)	5	4 (80%)
	GFP & AmCyan	4	4 (100%)
	Undetermined	5	4 (80%)
	Total	19	15 (79%)
Sorted for brightness	GFP (488/530nm)		4
Sorted for spectral shifts	GFP (488/530nm)		3
	AmCyan (405/525nm)		10

Supplementary Table 5. Individually expressed and tested designs.

RosettaScripts script and flags for modeling combinations of mutations. Every combination of mutations was modelled using a command based on:

```
rosetta_scripts_executable -database path_to_database -pdb_gz -overwrite -use_input_sc -
extrachi_cutoff 5 -ignore_unrecognized_res
-chemical:exclude_patches LowerDNA UpperDNA Cterm_amidation SpecialRotamer VirtualBB ShoveBB
VirtualDNAPhosphate VirtualNTerm CTermConnect sc_orbitals pro_hydroxylated_case1
pro_hydroxylated_case2 ser_phosphorylated thr_phosphorylated tyr_phosphorylated tyr_sulfated
lys_dimethylated lys_monomethylated lys_trimethylated lys_acetylated glu_carboxylated
cys_acetylated tyr_diiodinated N_acetylated C_methylamidated MethylatedProteinCterm -linmem_ig 10
-ignore_zero_occupancy false -no_nstruct_label true -in:file:native refined_pdb -extra_res_fa
LG.params -nstruct 30 -out:prefix NAME_
-s refined_pdb -use_occurrence_data -parser:protocol mutate_all_poss.xml -mute all -
parser:script_vars res_to_fix=94A,96A,121A,148A,203A,205A,222A,1X cst_full_path=ref_coord.cst
ignore_pose_profile_length_mismatch=1 min_aa_probability=-2 keep_n=1
all_ress=14A,16A,18A,42A,44A,46A,61A,64A,68A,69A,72A,108A,110A,112A,119A,123A,145A,150A,163A,165A
,167A,181A,185A,201A,220A,224A,42A,44A,61A,62A,69A,92A,94A,96A,112A,121A,145A,148A,150A,163A,165A
,167A,181A,183A,185A,203A,205A,220A,222A,224A -parser:script_vars target1=POS
new_res1=3_LETTER_AA
```

Where every mutation is listed as a separate target# and new_res#, the LG.params files is a parameters file a small ligand, in GFP’s case, it is the chromophore. The script is:

```
<ROSETTASCRIPTS>
    <SCOREFXNS>
        <ScoreFunction name="scorefxn_full" weights="ref2015">
            <Reweight scoretype="coordinate_constraint" weight="0.1"/>
        </ScoreFunction>
        <ScoreFunction name="soft_rep_full" weights="soft_rep">
            <Reweight scoretype="coordinate_constraint" weight="0.1"/>
        </ScoreFunction>
    </SCOREFXNS>
    <RESIDUE_SELECTORS>
            <Index name="ress_fix" resnums="%%res_to_fix%%"/>
    </RESIDUE_SELECTORS>
    <TASKOPERATIONS>
        <RestrictToRepacking name="rtr"/>
        <OperateOnResidueSubset name="fix_not_neighbor">
            <Not>
            <Neighborhood distance="8">
                    <Index resnums="%%all_ress%%"/>
            </Neighborhood>
            </Not>
            <PreventRepackingRLT/>
        </OperateOnResidueSubset>
        <InitializeFromCommandline name="init"/>
        <IncludeCurrent name="include_curr"/>
        <OperateOnResidueSubset name="fix_res" selector="ress_fix">
                <PreventRepackingRLT/>
        </OperateOnResidueSubset>
        <OperateOnResidueSubset name="not_to_cst_sc">
            <Not selector="ress_fix"/>
            <PreventRepackingRLT/>
```

```
        </OperateOnResidueSubset>
    </TASKOPERATIONS>
    <FILTERS>
        <DesignableResidues name="designable" task_operations="fix_not_neighbor" designable="0"
packable="1"/>
    </FILTERS>
    <MOVERS>
          <MutateResidue name="mutres0" new_res="%%new_res0%%" target="%%target0%%"
preserve_atom_coords="1"/>
        <ConstraintSetMover name="add_CA_cst" cst_file="%%cst_full_path%%"/>
        <AtomCoordinateCstMover name="fix_res_sc_cst" coord_dev="0.5" bounded="false"
sidechain="true" task_operations="not_to_cst_sc"/>
        <PackRotamersMover name="prm"
task_operations="init,include_curr,rtr,fix_not_neighbor,fix_res" scorefxn="scorefxn_full"/>
        <RotamerTrialsMinMover name="rtmin"
task_operations="init,include_curr,rtr,fix_not_neighbor,fix_res" scorefxn="scorefxn_full"/>
        <MinMover name="min" bb="1" chi="1" jump="1" scorefxn="scorefxn_full"/>
        <PackRotamersMover name="soft_repack" scorefxn="soft_rep_full"
task_operations="init,include_curr,rtr,fix_not_neighbor,fix_res"/>
    </MOVERS>
    <PROTOCOLS>
        <Add mover="add_CA_cst"/>
        <Add mover="fix_res_sc_cst"/>
        <Add mover="mutres0"/>
        <Add mover="soft_repack"/>
        <Add mover="min"/>
        <Add mover="prm"/>
        <Add mover="min"/>
        <Add filter="designable"/>
    </PROTOCOLS>
    <OUTPUT scorefxn="scorefxn_full"/>
</ROSETTASCRIPTS>
```
