# Supplementary material for: Designed active-site library reveals thousands of functional GFP variants
Source: Nat Commun. 2023 May 20;14:2890. doi: 10.1038/s41467-023-38099-z (PMC10199939; doi:10.1038/s41467-023-38099-z)
Supplement: Supplementary file 3 — Description of Additional Supplementary Files [file 41467_2023_38099_MOESM3_ESM.pdf]

Description of Additional Supplementary Files:

Supplementary Dataset 1. nohbonds library neighborhoods.

Supplementary Dataset 2. hbonds library neighborhoods.

Supplementary Dataset 3. Amino acids after filtration and EpiNNet enrichment for the nohbonds library.

A) each position has, at a minimum, a single amino acid, the PROSS-eGFP identity at that position.

B) position 65 is part of the chromophore and was mutated to Ser without being modelled in the Rosetta calculations.

Supplementary Dataset 4. Amino acids after filtration and EpiNNet enrichment for the hbonds library.

A) each position has, at a minimum, a single amino acid, the PROSS-eGFP identity at that position.

B) position 65 is part of the chromophore and was mutated to Ser without being modelled in the Rosetta calculations.

Supplementary Dataset 5. Sequence spaces of the two libraries.

Supplementary Dataset 6. Mutations occurring functional sequences from both libraries.

Supplementary Dataset 7. Biophysical characterization of the individually tested designs.

Source data are provided as a Source Data file.

Supplementary Dataset 8. DNA oligos required to clone the libraries
