# Supplementary material for: Designed active-site library reveals thousands of functional GFP variants
Source: Nat Commun. 2023 May 20;14:2890. doi: 10.1038/s41467-023-38099-z (PMC10199939; doi:10.1038/s41467-023-38099-z)
Supplement: Supplementary file 12 — Reporting Summary [file 41467_2023_38099_MOESM12_ESM.pdf]

nature portfolio

Corresponding author(s): Sarel Jacob Fleishman

Last updated by author(s): 03/17/2023

Reporting Summary

Nature Portfolio wishes to improve the reproducibility of the work that we publish. This form provides structure for consistency and transparency in reporting. For further information on Nature Portfolio policies, see our Editorial Policies and the Editorial Policy Checklist.

Statistics

For all statistical analyses, confirm that the following items are present in the figure legend, table legend, main text, or Methods section.

n/a	Confirmed	
☐	☒	The exact sample size (n) for each experimental group/condition, given as a discrete number and unit of measurement
☐	☒	A statement on whether measurements were taken from distinct samples or whether the same sample was measured repeatedly
☒	☐	The statistical test(s) used AND whether they are one- or two-sided<br>*Only common tests should be described solely by name; describe more complex techniques in the Methods section.*
☒	☐	A description of all covariates tested
☒	☐	A description of any assumptions or corrections, such as tests of normality and adjustment for multiple comparisons
☐	☒	A full description of the statistical parameters including central tendency (e.g. means) or other basic estimates (e.g. regression coefficient) AND variation (e.g. standard deviation) or associated estimates of uncertainty (e.g. confidence intervals)
☒	☐	For null hypothesis testing, the test statistic (e.g. F, t, r) with confidence intervals, effect sizes, degrees of freedom and P value noted<br>*Give P values as exact values whenever suitable.*
☒	☐	For Bayesian analysis, information on the choice of priors and Markov chain Monte Carlo settings
☒	☐	For hierarchical and complex designs, identification of the appropriate level for tests and full reporting of outcomes
☐	☒	Estimates of effect sizes (e.g. Cohen's d, Pearson's r), indicating how they were calculated

Our web collection on statistics for biologists contains articles on many of the points above.

Software and code

Policy information about availability of computer code

Data collection	All Rosetta design simulations used git version d9d4d5dd3fd516db1ad41b302d147ca0ccd78abd of the Rosetta biomolecular modeling software, which is freely available to academics at http://www.rosettacommons.org.<br>FACS data was collected using the FACS Diva software.
Data analysis	1. The algorithms for designing all libraries and for the random forest and epistasis analyses are available on GitHub<br>2. Deep-sequencing data was analyzed using the LAST package, version 1243<br>3. The random forest model was from the LightGBM model, version 3.2.1.99<br>4. all machine-learning related tools were from scikit-learn, version 0.24.2.

For manuscripts utilizing custom algorithms or software that are central to the research but not yet described in published literature, software must be made available to editors and reviewers. We strongly encourage code deposition in a community repository (e.g. GitHub). See the Nature Portfolio guidelines for submitting code & software for further information.

Data

Policy information about availability of data

All manuscripts must include a data availability statement. This statement should provide the following information, where applicable:

- Accession codes, unique identifiers, or web links for publicly available datasets
- A description of any restrictions on data availability
- For clinical datasets or third party data, please ensure that the statement adheres to our policy

All data will be available on GitHub alongside the code. sequences and data of individually tested designs are in Supplementary Dataset 7.

Human research participants

Policy information about studies involving human research participants and Sex and Gender in Research.

Reporting on sex and gender	This research did not involve human participants
Population characteristics	This research did not involve human participants
Recruitment	This research did not involve human participants
Ethics oversight	This research did not involve human participants

Note that full information on the approval of the study protocol must also be provided in the manuscript.

Field-specific reporting

Please select the one below that is the best fit for your research. If you are not sure, read the appropriate sections before making your selection.

[x] Life sciences [ ] Behavioural & social sciences [ ] Ecological, evolutionary & environmental sciences

For a reference copy of the document with all sections, see nature.com/documents/nr-reporting-summary-flat.pdf

Life sciences study design

All studies must disclose on these points even when the disclosure is negative.

Sample size	reported in all figures.
Data exclusions	no data was excluded, except in the photostability measurements, where some measurements were too noisy to fit a curve to.
Replication	photostability measurements were done with 4 replicates.<br>fluorescent lifetime were conducted with 3 replicates.<br>quantum yield and fluorescent spectra measurement were done in 3 replicates.<br>pH sensitivity measurements were done in 2 biological repeats with 3 technical repeats each.<br>thermostability was measure in 3 replicates.
Randomization	Randomization was not relevant to our study.
Blinding	Blinding was not relevant to our study.

Reporting for specific materials, systems and methods

We require information from authors about some types of materials, experimental systems and methods used in many studies. Here, indicate whether each material, system or method listed is relevant to your study. If you are not sure if a list item applies to your research, read the appropriate section before selecting a response.

Materials & experimental systems

n/a	Involved in the study
☒	☐ Antibodies
☒	☐ Eukaryotic cell lines
☒	☐ Palaeontology and archaeology
☒	☐ Animals and other organisms
☒	☐ Clinical data
☒	☐ Dual use research of concern

Methods

n/a	Involved in the study
☒	☐ ChIP-seq
☐	☒ Flow cytometry
☒	☐ MRI-based neuroimaging

Flow Cytometry

Plots

Confirm that:

☒ The axis labels state the marker and fluorochrome used (e.g. CD4-FITC).

☒ The axis scales are clearly visible. Include numbers along axes only for bottom left plot of group (a 'group' is an analysis of identical markers).

☒ All plots are contour plots with outliers or pseudocolor plots.

☒ A numerical value for number of cells or percentage (with statistics) is provided.

Methodology

Sample preparation	e. coli BL-21 cells were transformed with pBAD palsmids containing the designed libraries, and resuspended in LB for overnight growth. cultures were used to inoculate 50ml 2YT and induced with 0.2% arabinose when reaching OD600=0.6. induced cultures were moved to 20C overnight. samples were centrifuged and resuspended in fresh 2YT and shaken at 4C overnight.<br>on the day of the FACS sort samples were retrieved from 4C, washed with PBS twice and resuspended in PBS. samples were kept on ice and sorted in PBS.
Instrument	BD FACSAriaIII model number 648282D8 (Becton Dickinson)
Software	BD FACSDiva Software v8.0.1
Cell population abundance	Over 80% of the sorted populations contained active GFP as determined by visual inspection and plate reader measurements.
Gating strategy	The bacteria population was gated by looking at a FSC-H Vs. SSC-H plot. A tight gate was drawn around the major population. By running empty medium the gate was verified to be in the low noise region. A threshold was set on FSC-H and SSC-H channels to exclude major noise from analysis.<br><br>Fluorescent gate of GFP+ bacteria was defined based on a bacteria sample not expressing GFP. Gates were drawn around the positive areas containing no backround noise. Bacteria expressing eGFP were used to compensate for the spillover of from the GFP channel into the AmCyan channel. Gating of bacteria expressing designs excited by 405nm and emitting at 525nm was drawn on a GFP vs AmCyan channels plot, using bacteria expressing eGFP as a negative control.

☒ Tick this box to confirm that a figure exemplifying the gating strategy is provided in the Supplementary Information.
